# Has Childhood Smoking Reduced Following Smoke-Free Public Places Legislation? A Segmented Regression Analysis of Cross-Sectional UK School-Based Surveys

**DOI:** 10.1093/ntr/ntw018

**Published:** 2016-02-24

**Authors:** Srinivasa Vittal Katikireddi, Geoff Der, Chris Roberts, Sally Haw

**Affiliations:** ^1^MRC/CSO Social and Public Health Sciences Unit, University of Glasgow, Glasgow, UK;; ^2^Social Research and Information Division, Welsh Government, Cardiff, UK;; ^3^School of Health Sciences, University of Stirling, Stirling, UK

## Abstract

**Introduction:**

Smoke-free legislation has been a great success for tobacco control but its impact on smoking uptake remains under-explored. We investigated if trends in smoking uptake amongst adolescents differed before and after the introduction of smoke-free legislation in the United Kingdom.

**Methods:**

Prevalence estimates for regular smoking were obtained from representative school-based surveys for the four countries of the United Kingdom. Post-intervention status was represented using a dummy variable and to allow for a change in trend, the number of years since implementation was included. To estimate the association between smoke-free legislation and adolescent smoking, the percentage of regular smokers was modeled using linear regression adjusted for trends over time and country. All models were stratified by age (13 and 15 years) and sex.

**Results:**

For 15-year-old girls, the implementation of smoke-free legislation in the United Kingdom was associated with a 4.3% reduction in the prevalence of regular smoking (*P* = .029). In addition, regular smoking fell by an additional 1.5% per annum post-legislation in this group (*P* = .005). Among 13-year-old girls, there was a reduction of 2.8% in regular smoking (*P* = .051), with no evidence of a change in trend post-legislation. Smaller and nonsignificant reductions in regular smoking were observed for 15- and 13-year-old boys (*P* = .175 and *P* = .113, respectively).

**Conclusions:**

Smoke-free legislation may help reduce smoking uptake amongst teenagers, with stronger evidence for an association seen in females. Further research that analyses longitudinal data across more countries is required.

**Implications:**

Previous research has established that smoke-free legislation has led to many improvements in population health, including reductions in heart attack, stroke, and asthma. However, the impacts of smoke-free legislation on the rates of smoking amongst children have been less investigated. Analysis of repeated cross-sectional surveys across the four countries of the United Kingdom shows smoke-free legislation may be associated with a reduction in regular smoking among school-aged children. If this association is causal, comprehensive smoke-free legislation could help prevent future generations from taking up smoking.

## Introduction

Comprehensive smoke-free legislation has been heralded as one of the great successes of tobacco control for a generation, with the four countries of the United Kingdom amongst the first jurisdictions worldwide to adopt it.^1,2^ Its implementation is associated with several health benefits including improvements in respiratory health in bar workers, reductions in hospital admission for myocardial infarctions, stroke, and asthma in the general population and a reduction in perinatal complications for both pregnant women and their babies.^3–7^ An extensive international literature has also documented the adverse health effects of exposure to secondhand smoke (SHS) in adults and children.^8–11^ Fetuses, infants and children are particularly susceptible. In children, SHS exposure increases the risk of sudden infant death, lower respiratory infections (particularly bronchiolitis), asthma and middle ear disease, as well as impairing lung function.

In addition to the direct health benefits, support for smoke-free legislation has been shown to increase following implementation and this has been accompanied by changing social norms about the acceptability both of smoking and of exposing others to SHS.^12,13^ In Scotland, there was an increase in quitting behavior around the implementation of the ban, followed by a short-term reduction in smoking prevalence in adults but this was not sustained beyond a year.^14^ Exposure of children to SHS in the home similarly decreased.^15,16^

Legislation in the four UK countries is similar and broadly meets the definition of comprehensive smoke-free legislation as recommended by the WHO FCTC Article 8 legislation, that is, a ban on smoking in all enclosed public places and workplaces, including bars, restaurants and public transportation.^17^ Within the United Kingdom, it was implemented first in Scotland in March 2006, then in Wales and Northern Ireland in April 2007 and in England the following July.^18^ The few exemptions include designated rooms in hotels, care homes and hospices across the United Kingdom.^8^ In UK adult prisons, smoking is not allowed in communal spaces, but has been allowed in cells and exercise yards. In Scotland and Wales mental health units are also exempted.^8^

Long-term smoking patterns are typically established in adolescence, with earlier uptake linked to heavier smoking and a lower likelihood of future quitting.^19–21^ Although smoke-free legislation has been introduced in over 100 countries, most have still not implemented comprehensive legislation. It is possible that comprehensive smoke-free legislation could play an important role in preventing smoking uptake but to our knowledge, this specific hypothesis has not previously been studied.

In this article, we investigate whether trends in smoking uptake amongst children (aged 13 and 15 years) differed before and after the introduction of the comprehensive smoking ban in the four UK countries.

## Methods

Data on smoking prevalence among 13- and 15-year-old school children (stratified by sex) were obtained for the four UK countries from four repeat cross sectional surveys: the Smoking, Drinking and Drug Use Among Young People in England survey (SDDYP), the Scottish Schools Adolescent Lifestyle and Substance Use Survey (SALSUS), the Health Behaviour in School-aged Children (HBSC) survey in Wales^22–24^ and the Young Persons’ Behaviour & Attitudes Survey (YPBAS) in Northern Ireland. The SDDYP and SALSUS are conducted biennially and were chosen in preference to the HBSC surveys in these countries as they are only conducted every 4 years. The HBSC in Wales was generally conducted every 2 years while the YPBAS in Northern Ireland was carried out every 3–4 years. Each survey series asked about smoking using similar questionnaire items, with regular smoking defined as smoking at least one cigarette per week. Children aged 13 and 15 years were studied, since data for these age groups were consistently available across the different surveys and over time. Data on regular smoking prevalence together with available information on sample sizes were extracted from published data. Agencies holding the data were also contacted for further data when required. Year of implementation of smoke-free legislation were entered into the dataset. Ethical approvals for data collection were obtained by survey organizers.

Data were analyzed using segmented linear regression.^25^ The percentage of regular smokers was the outcome and each annual prevalence estimate from each country contributed one data point. Linear and quadratic terms in time were included to account for secular trends in smoking prevalence. The immediate effect of the intervention was modeled by a dummy variable indicating whether the observation was pre- or post-intervention. To allow for a change in trend in the years following the intervention an additional term, time post intervention, was included. Time invariant differences between countries were modeled as a set of dummy variables with England as the reference country.

Our statistical model is, therefore:

$$\text{Prevalence }=\alpha +\beta_{1}\text{time }+\beta_{2}{\text{time}}^{2}+\beta_{3}\text{interv }+\beta_{4}\text{postslope }+\beta_{\text{i}}{\text{country}}_{\text{i}}+\varepsilon$$

where time and time^2^ are linear and quadratic terms in years to account for secular trends; interv equals 0 pre-intervention and 1 for the year of intervention onwards; postslope is a count variable of years which starts at 1 from the intervention implementation year but is 0 prior to the intervention; and ε is an error term.

All models were stratified by age (13 and 15 years) and sex. Analyses were run using Stata SE 13.1.

## Results

Data were available for 55 time points for the four UK countries (see Supplementary Tables 1 and 2 for full data by country, year, age and sex). A downward trend in recent years was noted for all four countries. For example, the prevalence in 15-year-old boys in England fell from 24% in 1982 to 8% in 2013; equivalent figures for 15-year-old girls in England were 25% to 8%. Across all countries and all years, the mean prevalence (and *SD*) of self-reported smoking was: 17.6% (*SD* 5.91) for 15-year-old boys; 23.1% (*SD* 5.99) for 15-year-old girls; 5.75% (*SD* 2.59) for 13-year-old boys; and 8.44% (*SD* 4.01) for 13-year-old girls.

**Figure 1.**
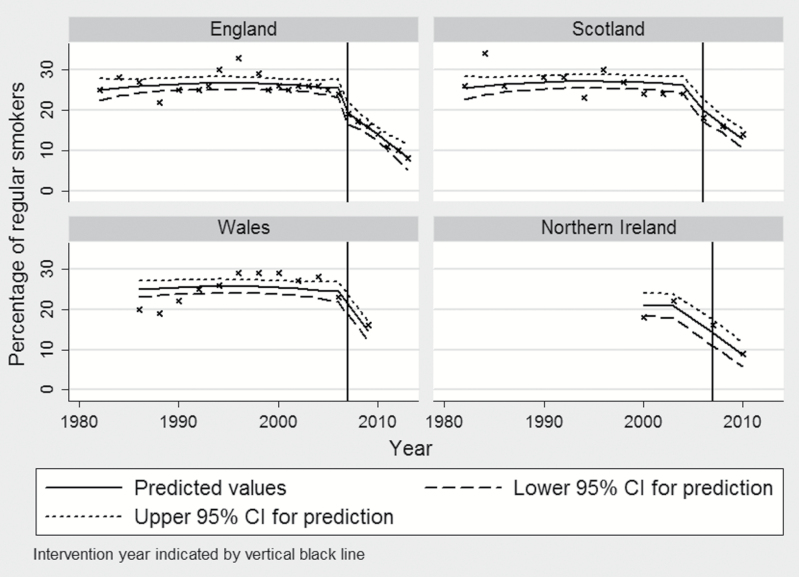
Model predictions for regular smoking amongst 15-year-old females in the four countries of the United Kingdom.

Table 1 shows associations between the introduction of smoke-free legislation and self-reported regular smoking from four separate regression models for each age-sex group (Figure 1 and Supplementary Figures 1–3 depict the results graphically).

**Table 1.** Associations Between Smoke-Free Public Places Legislation and Regular Smoking in Adolescents

	Males				Females			
	β	*P*	Lower 95% CI	Upper 95% CI	β	*P*	Lower 95% CI	Upper 95% CI
15-year olds								
Step-change	−3.527	.175	−8.681	1.628	−4.307	.029	−8.162	−0.452
Trend change	0.137	.842	−1.240	1.514	−1.516	.005	−2.546	−0.486
Time	0.005	.988	−0.687	0.697	0.275	.291	−0.243	0.792
Time^2^	−0.011	.386	−0.036	0.014	−0.010	.284	−0.029	0.009
England	REF	REF	REF	REF	REF	REF	REF	REF
Scotland	0.905	.475	−1.625	3.435	0.419	.658	−1.473	2.311
Wales	−3.205	.020	−5.879	−0.532	−0.988	.325	−2.987	1.012
Northern Ireland	−5.439	.011	−9.561	−1.317	−5.155	.002	−8.237	−2.072
*R* ^2^	0.6616				0.8157			
13-year olds								
Step-change	−1.634	.113	−3.666	0.399	−2.756	.051	−5.524	0.011
Trend change	0.078	.773	−0.465	0.621	−0.126	.733	−0.866	0.613
Time	−0.039	.777	−0.312	0.234	0.384	.043	0.013	0.756
Time^2^	−0.003	.518	−0.013	0.007	−0.013	.056	−0.027	0.000
England	REF	REF	REF	REF	REF	REF	REF	REF
Scotland	1.670	.002	0.672	2.668	1.505	.031	0.147	2.864
Wales	2.358	.000	1.304	3.412	5.763	.000	4.328	7.198
Northern Ireland	0.257	.752	−1.368	1.883	0.550	.619	−1.663	2.764
*R* ^2^	0.7262				0.7884			

CI = confidence interval; REF = reference group.

We found that implementation of the smoke-free legislation was followed by a reduction in regular smoking but, the reduction was only statistically significant for 15-year-old girls. In this group, implementation of smoke-free legislation was associated with a reduction in regular smoking by 4.3% (95% confidence interval [CI] −8.1, −0.45; *P* = .029). In addition, the prevalence of regular smoking fell by an additional 1.5% per annum post-legislation in this group (95% CI −2.5, −0.49; *P* = .005). Among 13-year-old girls, the prevalence of regular smoking reduced by 2.8% but this change just failed to reach statistical significance (95% CI −5.5, 0.011; *P* = .051). There was no evidence of a change in trend post legislation in this age-sex group.

Boys also experienced reductions in the prevalence of regular smoking, but these changes were smaller than their female peers and not statistically significant. Among 15-year-old boys, smoking prevalence fell by 3.5% (95% CI −8.7, +1.6; *P* = .175) while 13-year-old boys experienced a reduction of 1.6% (95% CI −3.7, +0.4; *P* = .113).

To ensure that our results were not unduly influenced by countries with few post-intervention data points only, we carried out sensitivity analysis of data from England only and found similar results (Supplementary Table 3).

## Discussion

In this study, we tested two a priori hypotheses: first, whether the prevalence of smoking amongst children at age 13 and age 15 differs before and after smoke-free legislation; and second, if the longer-term trend in smoking amongst children changed following the policy. The results from our study suggest that the implementation of smoke-free legislation may have a differential effect on boys and girls in the UK countries. Declines in smoking prevalence amongst school children were associated with the introduction of comprehensive smoke-free legislation within the different UK countries, but only reached statistical significance for 15-year-old females. The results are consistent with a reduction in initiation of smoking amongst the other age-sex groups (as indicated by the direction of effect, with wide 95% CIs), but there was limited statistical power due to few post-observation time points.

Our analysis is based on data which are intended to be representative of school-aged children in the four countries of the United Kingdom. However, some limitations should be noted. First, this analysis cannot establish causality between the introduction of smoke-free legislation and changes in children’s smoking uptake but rather only demonstrates an association. Second, while we have taken care to establish the comparability of questions between countries and over time, survey methods and response rates varied. Third, we have limited power to detect changes in longer-term trends following the intervention and repeating this analysis after more time has elapsed may be helpful. It is possible that future analyses may find similar associations exist amongst males—once adequate power is available to detect smaller effects. Fourth, we lacked information on potentially important covariates (eg, parental home smoking, youth working) which would have allowed better investigation of the legislation’s impact. Lastly, it is possible other policies or interventions may have been introduced around the time of smoke-free legislation (eg, increases in the legal purchasing age) and were responsible for the observed changes. However, disentangling causality may be particularly problematic since smoke-free legislation could have indirectly contributed to the implementation of other interventions by encouraging awareness and debate about tobacco control amongst policymakers and the general public which in turn produced a facilitative environment for tobacco control. For example, smoke-free legislation in public places in Spain was found to encourage tobacco control activities in hospitals.^26^

There is evidence smoke-free legislation may help trigger smoking cessation for some people, but the effect appears short-lived.^27^ However, an effect on smoking initiation amongst adolescents would potentially have long-lasting benefits for future generations. Previous research found smoke-free environments in general are associated with reduced likelihood of teenagers initiating smoking,^28,29^ suggesting an effect is plausible. Indeed, qualitative evidence reported smoke-free legislation itself may result in families avoiding smoking in the home or in front of children, assisting in denormalization of smoking behaviour.^30^ The public debate that frequently accompanies the introduction of smoke-free legislation may act as an important trigger in this regard. Ethnographic evidence also exists for young people’s subcultures to shift in response to smoke-free legislation,^31^ making night-time environments (such as pubs/nightclubs) also worthy of investigation. The comprehensive nature of the legislation may therefore serve both to denormalize smoking and alter social environments. This may make the initiation of smoking less likely and could therefore provide the mechanisms by which smoke-free legislation has an effect on adolescent uptake. It is possible the impact of certain types of environment on males and females may differ,^32^ but this hypothesis needs further examination.

Smoking is an important contributor to gender differences in mortality, accounting for 40% to 60% of the gender gap, but concerns exist that the future burden in females will increase substantially.^33^ It is possible smoke-free legislation may reduce smoking uptake amongst girls which has important implications for gender equity in the future tobacco epidemic. However, a study of the Spanish experience with smoke-free policy found no effect on smoking prevalence in 15–24-year-olds, with no difference by gender.^34^

UK smoke-free legislation has previously been found to result in significant declines in SHS in primary school children but the limited available evidence suggests it may have widened inequalities in exposure.^35,36^ Our results suggest smoke-free legislation may help reduce smoking uptake amongst teenagers, as well as reduce the harmful effects of SHS and triggering smoking cessation. However, investigating impacts on equity of this potential effect is important, since lifelong impacts on inequalities could occur.

Our study provides, to our knowledge, the first evidence to suggest that smoke-free legislation could reduce smoking uptake. Further research analyzing longitudinal data across more countries is required to establish if the association observed is causal. Cross-national comparative research may help determine which aspects of implementation are associated with the greatest reductions in smoking uptake amongst children.

## Supplementary Material

Supplementary Tables 1–3 and Figures 1–3 can be found online at http://www.ntr.oxfordjournals.org

## Funding

This study received no dedicated funding. SVK was funded by the Chief Scientist Office at the Scottish Health Directorates as part of the of the Evaluation of Social Interventions programme at the MRC/CSO Social & Public Health Sciences Unit (MC_UU_12017/4). GD was funded by the Medical Research Council as part of the Social Patterning of Health over the Lifecourse programme (MC_UU_12017/5).

## Declaration of Interests

*None declared.*

## Supplementary Material

Supplementary Data
