# Supplementary material for: Has Childhood Smoking Reduced Following Smoke-Free Public Places Legislation? A Segmented Regression Analysis of Cross-Sectional UK School-Based Surveys
Source: Nicotine Tob Res. 2016 Feb 24;18(7):1670–4. doi: 10.1093/ntr/ntw018 (PMC4902887; doi:10.1093/ntr/ntw018)
Supplement: Supplementary Data [file supp_18_7_1670__index.html]

Has Childhood Smoking Reduced Following Smoke-Free Public Places Legislation? A Segmented Regression Analysis of Cross-Sectional UK School-Based Surveys — Has Childhood Smoking Reduced Following Smoke-Free Public Places Legislation? A Segmented Regression Analysis of Cross-Sectional UK School-Based Surveys — Supplementary Data 

# Has Childhood Smoking Reduced Following Smoke-Free Public Places Legislation? A Segmented Regression Analysis of Cross-Sectional UK School-Based Surveys

## Supplementary Data

Data files

- Supplementary Data - Supplementary Data
