# Supplementary material for: Has Childhood Smoking Reduced Following Smoke-Free Public Places Legislation? A Segmented Regression Analysis of Cross-Sectional UK School-Based Surveys
Source: Nicotine Tob Res. 2016 Feb 24;18(7):1670–4. doi: 10.1093/ntr/ntw018 (PMC4902887; doi:10.1093/ntr/ntw018)
Supplement: Supplementary Data [file supp_ntw018_NicTobRes_SuppMaterial_v3a.doc]

**SUPPLEMENTARY MATERIAL**

**Supplementary Table 1: Availability of survey data**

| **Country** | **Years of data availability** |
| --- | --- |
| England | 1982, 1984, 1986, 1988, 1990, 1992, 1994, 1996, 1998, 1999, 2000, 2001, 2002, 2003, 2004, 2005, 2006, 2007, *2008, 2009, 2010, 2011, 2012, 2013* |
| Northern Ireland | 2000, 2003, 2007, *2010* |
| Scotland | 1982, 1984, 1986, 1988, 1990, 1992, 1994, 1996, 1998, 2000, 2002, 2004, 2006, 2008, *2010* |
| Wales | 1986, 1988, 1990, 1992, 1994, 1996, 1998, 2000, 2002, 2004, 2006, *2009* |

Italics indicate post-intervention data points.

Data were from the Smoking, Drinking and Drug Use Among Young People in England survey (SDDYP), the Scottish Schools Adolescent Lifestyle and Substance Use Survey (SALSUS), the Health Behaviour in School-aged Children (HBSC) survey in Wales; and the Young Persons’ Behaviour & Attitudes Survey (YPBAS) in Northern Ireland. The same survey series was used for each country over time, given the differences in survey methodology. Between countries, differences in survey methodology should be accounted for by the fixed-effects analysis.

All four survey series are designed to be representative of the schoolchildren for their respective countries and are conducted for the purposes of monitoring trends in health-related behaviours. Consistent methods have been used over time, to maintain comparability within each country over time. Extensive documentation for each of these surveys has been published and is available through the references cited.

In brief, each survey uses multi-stage sampling, with the primary sampling unit being schools. Schools are selected through stratified probability samples, to ensure adequate representation of different geographical regions and all pupils attending school on a single day are asked to complete the survey during school hours. Surveys are self-completed anonymously, with demographic information provided by the pupils. Smoking status was ascertained through self-report.

In England and Scotland, the following question was used:

“Now read the following statements carefully and cross the box next to the one which best

describes you:

- I have never smoked
- I have only ever tried smoking once
- I used to smoke sometimes but I never smoke a cigarette now
- I sometimes smoke cigarettes now but I don’t smoke as many as one a week
- I usually smoke between one and six cigarettes a week
- I usually smoke more than six cigarettes a week”

In Northern Ireland, regular smoking of at least once per week was ascertained through the following question:

“How often do you smoke cigarettes now?

- Every day
- At least once a week but not every day
- Less than once a week
- I do not smoke now”

In Wales, smoking status was determined by asking:

“How often do you smoke tobacco at present?

- Every day
- At least once a week, but not every day
- Less than once a week
- I do not smoke”

Trends in regular smoking (i.e. at least once per week) have been reported by each of the survey teams and these data are collated below. Given the yearly availability of data, there is theoretically the potential for misclassification of pre- and post-intervention data. However, since most surveys are not available in the year of implementation, the importance of this is likely to be minor. Furthermore, the impact of such misclassification is likely to result in an underestimation of the magnitude of the association between the policy and smoking prevalence.

**Supplementary T**able 2: Prevalence of regular smoking from UK school-based surveys

|  |  | % regular smokers (at least once per week) | | | |
| --- | --- | --- | --- | --- | --- |
| Year | Country | 15 yr old males | 15 yr old females | 13 yr old males | 13 yr old females |
| 1982 | England | 24 | 25 | 8 | 6 |
| 1984 | England | 28 | 28 | 10 | 9 |
| 1986 | England | 18 | 27 | 5 | 5 |
| 1988 | England | 17 | 22 | 5 | 4 |
| 1990 | England | 25 | 25 | 6 | 9 |
| 1992 | England | 21 | 25 | 6 | 9 |
| 1993 | England | 19 | 26 | 3 | 5 |
| 1994 | England | 26 | 30 | 4 | 8 |
| 1996 | England | 28 | 33 | 8 | 11 |
| 1998 | England | 19 | 29 | 5 | 9 |
| 1999 | England | 21 | 25 | 4 | 8 |
| 2000 | England | 21 | 26 | 6 | 10 |
| 2001 | England | 19 | 25 | 5 | 8 |
| 2002 | England | 20 | 26 | 5 | 8 |
| 2003 | England | 18 | 26 | 5 | 8 |
| 2004 | England | 16 | 26 | 5 | 6 |
| 2005 | England | 16 | 25 | 5 | 6 |
| 2006 | England | 16 | 24 | 3 | 7 |
| 2007 | England | 12 | 19 | 3 | 4 |
| 2008 | England | 11 | 17 | 3 | 6 |
| 2009 | England | 14 | 16 | 3 | 4 |
| 2010 | England | 10 | 14 | 3 | 4 |
| 2011 | England | 11 | 11 | 2 | 3 |
| 2012 | England | 10 | 10 | 1 | 2 |
| 2013 | England | 8 | 8 | 1 | 1 |
| 1982 | Scotland | 29 | 26 | 8 | 10 |
| 1984 | Scotland | 29 | 34 | 10 | 12 |
| 1986 | Scotland | 24 | 26 | 7 | 8 |
| 1990 | Scotland | 22 | 28 | 8 | 10 |
| 1992 | Scotland | 22 | 28 | 9 | 9 |
| 1994 | Scotland | 20 | 23 | 11 | 10 |
| 1996 | Scotland | 30 | 30 | 8 | 10 |
| 1998 | Scotland | 25 | 27 | 9 | 11 |
| 2000 | Scotland | 15 | 24 | 5 | 10 |
| 2002 | Scotland | 16 | 24 | 6 | 9 |
| 2004 | Scotland | 14 | 24 | 5 | 7 |
| 2006 | Scotland | 12 | 18 | 3 | 5 |
| 2008 | Scotland | 14 | 16 | 3 | 4 |
| 2010 | Scotland | 11 | 14 | 3 | 3 |
| 1986 | Wales | 16 | 20 | 7 | 12 |
| 1988 | Wales | 12 | 19 | 9 | 11 |
| 1990 | Wales | 14 | 22 | 8 | 11 |
| 1992 | Wales | 18 | 25 | 10 | 15 |
| 1994 | Wales | 18 | 26 | 8 | 13 |
| 1996 | Wales | 23 | 29 | 11 | 16 |
| 1998 | Wales | 21 | 29 | 8 | 19 |
| 2000 | Wales | 20 | 29 | 6 | 17 |
| 2002 | Wales | 15 | 27 | 8 | 15 |
| 2004 | Wales | 19 | 28 | 8 | 13 |
| 2006 | Wales | 12 | 23 | 6 | 13 |
| 2009 | Wales | 11 | 16 | 3 | 6 |
| 2000 | N Ireland | 8 | 18 | 5 | 13 |
| 2003 | N Ireland | 15 | 22 | 4 | 5 |
| 2007 | N Ireland | 7 | 16 | 2 | 5 |
| 2010 | N Ireland | 10 | 9 | 4 | 2 |

**Supplementary Figure 1: Model predictions for regular smoking amongst 15 year old males in the four countries of the UK**


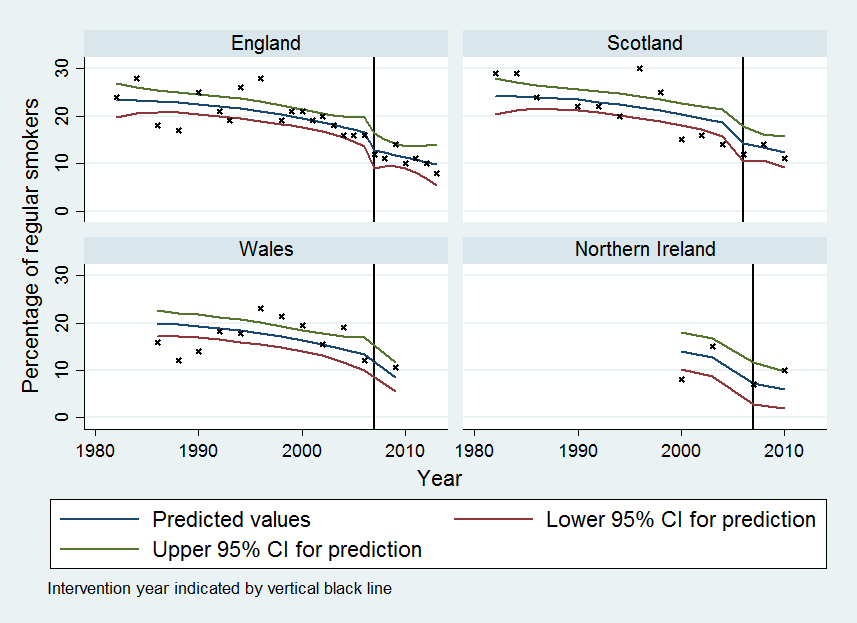


**Supplementary Figure 2: Model predictions for regular smoking amongst 13 year old males in the four countries of the UK**


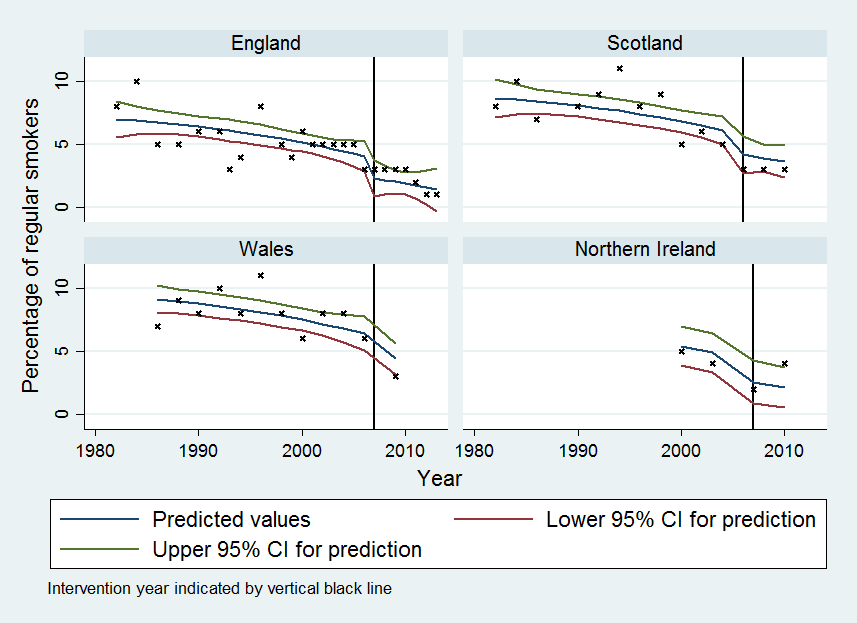


**Supplementary Figure 3: Model predictions for regular smoking amongst 13 year old females in the four countries of the UK**


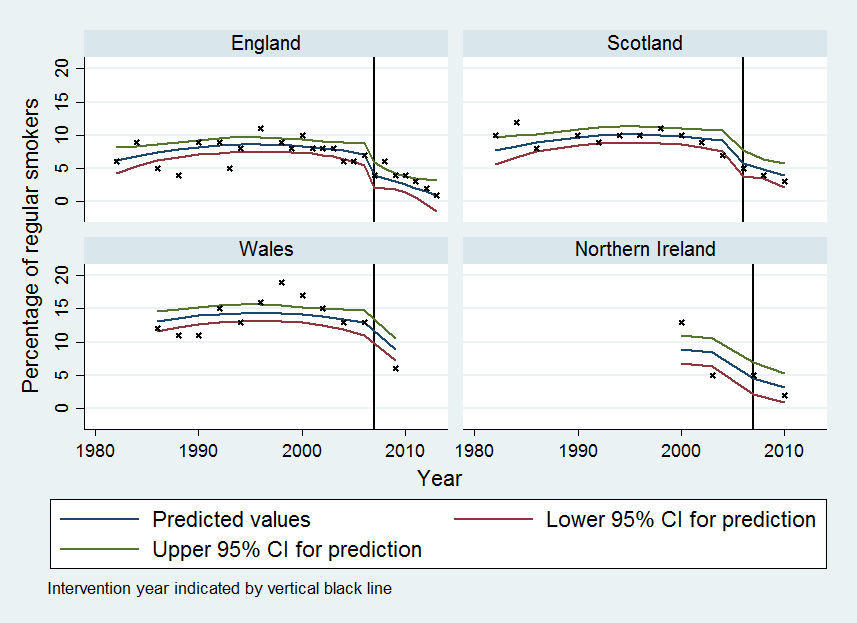


**Supplementary Table 3: Models repeated using** English data only

|  | **Males** | | | | **Females** | | | |
| --- | --- | --- | --- | --- | --- | --- | --- | --- |
|  | beta | p | Lower 95% CI | Upper 95% CI | beta | p | Lower 95% CI | Upper 95% CI |
| **15 year olds** | | | | | | | | |
| step-change | -3.077 | 0.322 | -9.403 | 3.248 | -3.905 | 0.082 | -8.357 | 0.548 |
| trend change | 0.318 | 0.676 | -1.247 | 1.883 | -1.285 | 0.024 | -2.387 | -0.184 |
| Time | 0.269 | 0.527 | -0.603 | 1.142 | 0.462 | 0.132 | -0.152 | 1.076 |
| Time^2^ | -0.021 | 0.189 | -0.052 | 0.011 | -0.018 | 0.110 | -0.040 | 0.004 |
| R^2^ | 0.7609 | | | | 0.9097 | | | |
| **13 year olds** | | | | | | | | |
| step-change | -0.840 | 0.534 | -3.610 | 1.931 | -1.004 | 0.548 | -4.425 | 2.418 |
| trend change | -0.523 | 0.127 | -1.208 | 0.163 | -0.228 | 0.581 | -1.075 | 0.619 |
| Time | -0.348 | 0.072 | -0.730 | 0.034 | 0.437 | 0.068 | -0.035 | 0.909 |
| Time^2^ | 0.008 | 0.228 | -0.006 | 0.022 | -0.015 | 0.088 | -0.032 | 0.002 |
| R^2^ | 0.7619 | | | | 0.6658 | | | |

Country omitted in the above models, since data are from only one country.
